# In Silico Identification of Novel Leads as Potential DPP-IV Inhibitors as Antidiabetic Agents Using Virtual Screening

**DOI:** 10.1021/acsomega.5c13626

**Published:** 2026-08-24

**Authors:** Milendra Kumar Turkar, Rahul Ahirwar, Rishika Sahu, Rajendra Bhadane, Surendra Kumar Jain, Deepti Jain

**Affiliations:** † School of Pharmaceutical Sciences, 74991 Rajiv Gandhi Proudyogiki Vishwavidyalaya, Airport Rd, Gandhi Nagar, Bhopal, Madhya Pradesh 462033, India; ‡ Institute of Biomedicine, Research Unit for Infection and Immunity, University of Turku, Kiinamyllynkatu 10, Turku FI-20520, Finland; § 74993 Lakshmi Narain College of Pharmacy, Kalchuri Nagar, Raisen Road, Bhopal 462021, India

## Abstract

**Purpose:** Dipeptidyl peptidase-IV (DPP-IV) is a validated therapeutic target for type 2 diabetes mellitus due to its role in incretin hormone degradation. This study aimed to identify novel small-molecule DPP-IV inhibitors from the ECBD database using an integrated virtual screening and molecular dynamics (MD) approach, acknowledging that experimental validation is necessary to confirm biological activity. **Methods:** Structure-based virtual screening of 5,500 ECBD compounds was performed using molecular docking to identify high-affinity ligands, followed by interaction analysis with key catalytic residues. Pharmacokinetic suitability was evaluated through in-silico ADMETox profiling. The top-ranked hits were further subjected to 100 ns MD simulations to assess complex stability and conformational effects on the DPP-IV active site. Binding free energies were calculated using the MM/GBSA method, and docking reliability was validated through redocking experiments. **Results and Discussion:** Five compounds EOS34295, EOS4915, EOS9480, EOS2567, and EOS62725 exhibited stronger noncovalent binding affinities than vildagliptin and formed stable interactions with essential residues, including GLU205, GLU206, ASN710, and PHE357. ADMETox predictions indicated favorable oral drug-likeness. MD simulations revealed stable protein–ligand complexes, with lower RMSD values for the hit ligands (1.20–1.51 Å) compared with vildagliptin (1.90 Å). RMSF analysis showed consistent flexibility without destabilizing fluctuations. Additional hydrogen-bond interactions with PHE357 emerged during MD simulations, indicating enhanced binding persistence. MM/GBSA analysis confirmed stronger binding energies for EOS34295 (−56.58 kcal/mol), EOS62725 (−37.90 kcal/mol), and EOS2567 (−31.27 kcal/mol) relative to vildagliptin (−23.88 kcal/mol). **Conclusion:** This study identifies EOS34295, EOS2567, and EOS62725 emerged as promising DPP-IV inhibitory hits with superior binding stability, supporting their potential as antidiabetic leads for future experimental validation.

## Graphical abstract

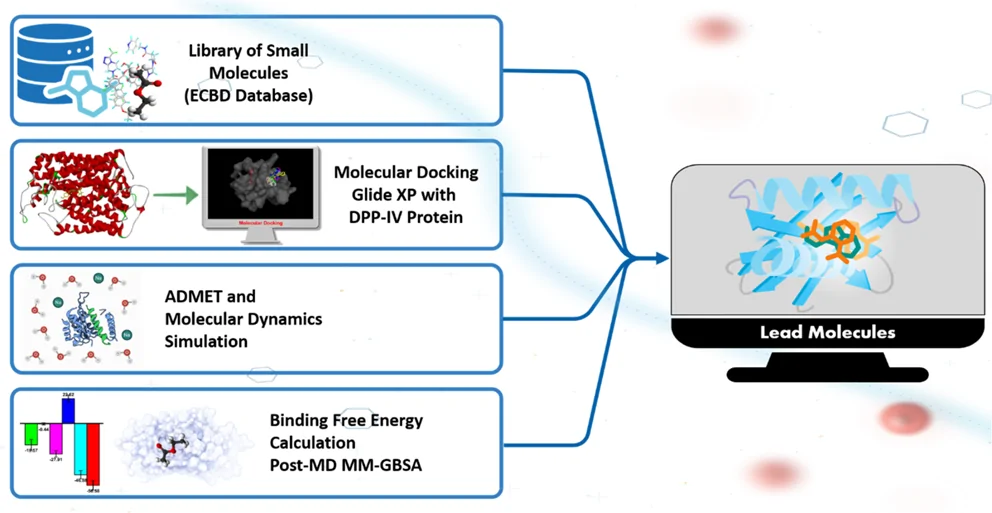

## 1 Introduction

Diabetes mellitus (DM) is a complex metabolic disorder marked by chronic hyperglycemia, which progressively damages vital organs including the eyes, kidneys, heart, blood vessels, and peripheral nerves.^1–3^ Over recent decades, diabetes has become one of the most rapidly expanding global health challenges.^4^ According to the International Diabetes Federation (IDF), approximately 1 in 11 adults worldwide is currently living with diabetes, with type 2 diabetes mellitus (T2DM) accounting for nearly 90% of all cases.^4–6^ The global burden continues to rise sharply, with an estimated 463 million individuals affected in 2019 and projections indicating an increase to 589 million by 2030 and nearly 700 million by 2045.^7,8^ Recent IDF reports^5^ also highlight that diabetes is responsible for roughly 10% of all deaths globally, underscoring both its prevalence and the severity of its long-term complications.^8,9^ T2DM develops primarily from inadequate insulin secretion combined with insulin resistance in peripheral tissues. Persistent hyperglycemia associated with T2DM leads to progressive dysfunction of multiple organs and contributes to long-term disability and diminished quality of life. Consequently, diabetes management has become a major healthcare challenge, placing significant physical, emotional, and financial burdens not only on affected individuals but also on families, healthcare systems, and broader communities.^10–12^ One of the most effective therapeutic strategies for T2DM focuses on the regulation of incretin hormones. Incretins including glucagon-like peptide-1 (GLP-1) and glucose-dependent insulinotropic polypeptide (GIP) are secreted by the intestine following nutrient intake and stimulate glucose-dependent insulin secretion from pancreatic β-cells.^13–17^ Their physiological activity, however, is short-lived because they are rapidly degraded by dipeptidyl peptidase IV (DPP-IV), a widely distributed serine protease.^18,19^ Consequently, inhibition of DPP-IV has become a validated target for T2DM therapy, as it prolongs the activity of endogenous incretins, thereby enhancing insulin release, reducing postprandial glucose levels, suppressing glucagon secretion, and improving glycemic control.^20,21^

Several DPP-IV inhibitors collectively known as gliptins have been approved for clinical use in the United States and Europe, including sitagliptin, saxagliptin, alogliptin, and vildagliptin (the latter approved in Europe). Although these medications have demonstrated significant therapeutic benefits, their clinical utility is constrained by adverse effects such as joint pain, pancreatitis, gastrointestinal disturbances, and an increased risk of heart failure.^22^ Despite their widespread use, these therapies may induce severe side effects, including cardiovascular complications, bone density loss, weight gain, edema, digestive disorders, and increased susceptibility to urinary tract infections. The limitations of existing drugs emphasize the urgent need for novel therapeutic agents that are more effective, safer, and economically accessible.^23–25^

In this context, computational drug-discovery approaches have emerged as powerful tools for accelerating the identification and optimization of new drug candidates. In silico methods such as virtual screening, molecular docking, ADMETox prediction, MD simulations, and binding free energy calculations using Molecular Mechanics-General Born Surface Area (MM-GBSA) offers rapid and cost-effective strategies to explore vast chemical spaces and predict interactions between candidate molecules and therapeutic targets with high precision. These approaches significantly reduce the experimental burden and enhance the likelihood of identifying promising lead compounds. In the present work (Figure 1) aims to identify potential DPP-IV inhibitors using a comprehensive computational workflow integrating structure-based virtual screening, ADMETox prediction, MD simulations, and MM-GBSA binding energy analysis. Screening was performed against the European Chemical Biology Database (ECBD),^26^ which contains over 106,000 structurally diverse compounds enriched in functionalized heterocycles, supported by data from 18 molecular targets and 30 validated biological assays.

**Figure 1:**
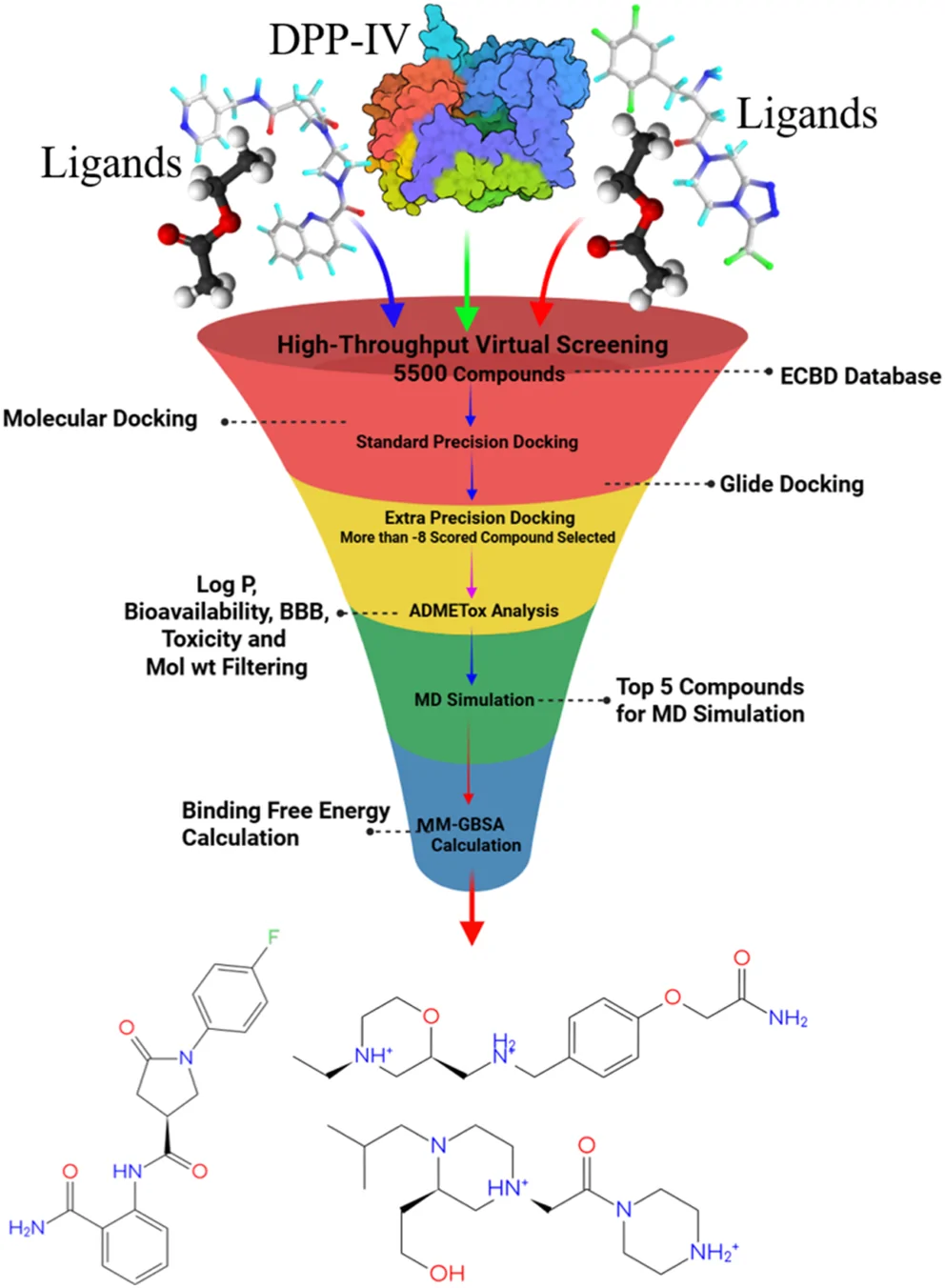
Workflow we adopted in this work for the identification of potential DPP-IV inhibitors.

Despite the availability of several computational studies on DPP-IV inhibitors, there remains a need for systematic and integrated screening approaches that combine large-scale virtual screening with dynamic and pharmacokinetic validation. In this context, the present study employs a multistep computational pipeline, including molecular docking, MM/GBSA binding free energy estimation, molecular dynamics simulations, and ADMET profiling, to identify and prioritize potential DPP-IV inhibitors with improved stability and drug-like properties.

This approach seeks to uncover novel ligands with strong inhibitory potential, favorable binding stability, and drug-like characteristics, contributing to the development of next-generation therapeutic candidates for the effective management of T2DM.

## 2 Material and Methods

### 2.1 Platform, Software, and Web Tools for Molecular Modeling

The computational studies including Molecular docking, MD simulation, binding free energy calculations, and visualization of the protein–ligand complexes were performed using Schrodinger drug discovery suite (Release 2022–4: Schrödinger) and PyMol version 2.3.0.

### 2.2 Database Selection and Ligand Preparation

To screen the potent and biologically viable compounds, we utilized ECBD^26^ (https://ecbd.eu/). The ECBD database is a reliable biological database containing high-quality chemical libraries for screening programs and is widely used in integrated high-throughput screening programs at pharmaceutical and biotechnological organizations. The library contains over 108856 compounds with functionalized heterocycles, 29 molecular targets, and 111 assays. In the present study, three-dimensional (3D) structures of the compounds downloaded from the ECBD database were imported into the Maestro molecular modeling suite. The stereochemical information available in the database entries was retained during the preparation process to preserve the structural integrity of the ligands. The compounds were filtered for drug-like properties which includes Lipinski’s rule of five. Figure 1 shows the virtual screening roadmap of the current study. The MacroModel^27^ tool of Maestro (Schrödinger Release 2022–4) was used to prepare and generate optimized, low-energy conformers of the ligands using the OPLS4 ^28^ During ligand preparation, hydrogen atoms were added and geometry optimization was performed to obtain energetically favorable conformations suitable for docking studies. For each compound possible tautomeric states were generated at a pH of 7.0 ± 2.0.

### 2.3 Protein Retrieval and Preparation

The crystal structure of human DPP-IV (PDB ID: 6B1E,^29^ 1.77 Å) was retrieved from the Protein Data Bank for use in molecular docking and MD simulations. Although both chains of the protein are cocrystallized with vildagliptin, structural inspection revealed poor resolution around SER680 in Chain A, obscuring the expected covalent bond with the ligand. In Chain B, however, the SER680–vildagliptin covalent linkage is clearly resolved, suggesting that the same interaction likely exists in Chain A but is not captured due to local resolution limitations. To enable accurate docking calculations, a noncovalent, unconstrained form of vildagliptin was regenerated and subsequently optimized through hydrogen-bond refinement and restrained minimization, resulting in slight adjustments in the orientation of the cyano and pyrrole moieties (Figure 3). This optimized ligand structure was used for protein preparation, receptor grid generation, and all downstream docking studies. Protein preparation was carried out using the Protein Preparation Wizard in Maestro (Schrödinger Release 2022–4). The workflow included assigning bond orders, adding missing hydrogen atoms, and optimizing the hydrogen-bonding network. Protonation states of ionizable residues were adjusted using PROPKA^30^ at pH 7.4 to reflect physiological conditions. Water molecules located more than 8 Å from the active site were removed, and the protein was subjected to restrained energy minimization using the OPLS4 ^28^ force field to relieve steric clashes while preserving the experimental geometry. The final minimized protein model, together with the optimized ligand, was used for receptor grid generation and subsequent virtual screening MD simulation analyses.

### 2.4 Molecular Docking Studies

Molecular docking and virtual screening are widely used computational approaches for predicting ligand binding orientations, identifying novel hits, and eliminating noncomplementary compounds within the binding pocket of a target protein. These methods serve as effective filtering tools in the early stages of drug discovery. In this study, the prepared structures of the target protein and ligand molecules were subjected to molecular docking using the Glide module of Maestro (Schrödinger Release 2022–4). Docking was carried out in extra-precision^31^ (XP) mode, which provides enhanced accuracy in pose prediction and scoring by employing a more rigorous sampling algorithm and scoring function. This approach enabled the identification of optimal binding conformations and offered detailed insights into key molecular interactions within the active site.

### 2.5 ADMETox Prediction

The pharmacokinetic and toxicity profiles of the top five compounds were evaluated using freely accessible computational web servers to obtain a comprehensive assessment of their drug-likeness and safety characteristics.

Physicochemical and ADME parameters were predicted using the SwissADME web server. The evaluated parameters included molecular weight (MW), fraction of sp^3^ carbons (Csp^3^), hydrogen bond donors (HBD), hydrogen bond acceptors (HBA), molar refractivity (MR), topological polar surface area (TPSA), lipophilicity (iLOGP), gastrointestinal (GI) absorption, blood–brain barrier (BBB) permeability, P-glycoprotein (P-gp) substrate prediction, cytochrome P450 inhibition profile (CYP1A2, CYP2C19, CYP2C9, and CYP3A4), skin permeability (log Kp), Lipinski rule violations, and bioavailability score. Lipinski’s rule of five criteria were used as a reference to assess oral drug-likeness. In addition, in-silico toxicity assessment was performed using the ProTox-II platform. Toxicological end points evaluated included hepatotoxicity, neurotoxicity, nephrotoxicity, respiratory toxicity, cardiotoxicity, immunotoxicity, mutagenicity, cytotoxicity, carcinogenicity, cytochrome inhibition potential, and predicted median lethal dose (LD_50_, mg/kg).

This combined ADME and toxicity evaluation enabled a systematic assessment of the pharmacokinetic suitability and safety profile of the identified lead compounds prior to further computational analyses.

### 2.6 MD Simulation

The protein–ligand complexes with the very good docking score were subjected for 100 ns MD simulations using the Desmond^32,33^ tool from Maestro. Before actual simulation the MD simulation system prepared using the system builder tool of Desmond with single point charge (SPC)^33,34^ water as an explicit solvation model and OPLS4 ^28^ force field. Each system was neutralized with appropriate numbers of Na^+^ or Cl^–^ ions, and a physiological salt concentration of 0.15 M NaCl was maintained. An orthorhombic simulation box, with Periodic Boundary Conditions (PBC) and a 10-Å buffer space between the solute and the box edge, was used for each system. Each system underwent energy minimization and brief equilibrium steps, including heating to 300 K and stabilizing at 1.01325 bar under NVT and NPT ensembles. These steps ensured the system reached equilibrium before entering the production simulations. The production simulations were conducted at temperature 300 K using the Nosé–Hoover chain thermostat,^35^ with a relaxation time of 1.0 ps. The pressure was set at 1.01325 bar with the Martyna–Tobias–Klein barostat,^36,37^ using isotropic coupling and a relaxation time of 2.0 ps. Long-range interactions were handled using the U-series method,^38^ and for short-range interactions, a cutoff radius of 9.0 Å was used. Postsimulation analyses were performed using the simulation interaction diagram to evaluate the trajectories, while graphical data representation was generated with Grace^39^ software. These analyses provided detailed insights into the dynamic behavior, stability, and interaction patterns of the protein–ligand complexes throughout the simulation.

### 2.7 Binding Free Energy Analysis

The binding free energy calculation provides a valuable predictive criterion to rank ligands based on their binding affinities toward a target protein. In this study, the MD trajectories of DPP-IV in complex with the selected compounds were subjected to postsimulation binding free energy analysis. The evaluation of the ligand binding free energy (Δ*G*_bind_, kcal/mol) was performed using the MM-GBSA module implemented in the Schrödinger suite (*thermal_mmgbsa.py*). Desmond generated MD trajectories with a total simulation length of 100 ns (200 frames) for each complex were used as input. The binding free energy values were computed based on standard energy terms, including van der Waals, Coulomb, solvation, lipophilic, and covalent contributions. The binding free energy was calculated using the following equations

$$\Delta G_{\mathrm{binding}}=G_{\mathrm{complex}}-\left(G_{\mathrm{protein}}+G_{\mathrm{ligand}}\right)$$

$$\Delta G_{\mathrm{bind}}=\Delta E_{\mathrm{MM}}+\Delta G_{\mathrm{solv}}+\Delta G_{\mathrm{SA}}$$

Where, Δ*E*_MM_ = total gas-phase energy from the OPL4 force field, consisting of Δ*E*_electrostatic_ (Coulomb interactions) and Δ*E*_vdW_ (van der Waals interactions), Δ*G*_solv_ = polar solvation free energy, estimated using the Generalized Born (GB) model, Δ*G*_SA_ = nonpolar solvation contribution (solvent-accessible surface area, SASA-based). The resulting Δ*G*_binding_ values were calculated across all 200 sampled frames, and the standard deviation was calculated using Microsoft Excel 2021 (Microsoft Corporation).

## 3 Results and Discussion

### 3.1 Virtual Screening and Molecular Docking

Extensive molecular docking studies were conducted to evaluate the binding interactions and inhibitory potential of newly identified compounds against the DPP-IV enzyme (PDB ID: 6B1E).^29^ A total of 5500 compounds were screened, with vildagliptin (the cocrystallized ligand) used as the reference standard. Docking results revealed a wide range of binding affinities from −4.00 to −12.26 kcal/mol. Among all screened molecules, five compounds demonstrated markedly superior binding affinity compared to vildagliptin (−7.58 kcal/mol). The top hits EOS34295 (−9.21 kcal/mol), EOS4915 (−8.41 kcal/mol), EOS9480 (−10.27 kcal/mol), EOS2567 (−10.54 kcal/mol), and EOS62725 (−12.26 kcal/mol) exhibited stronger predicted interactions with the DPP-IV active site. Superimposition of these hits with the standard ligand confirmed that they occupy the same catalytic pocket, supporting their potential as competitive inhibitors. The binding affinities of these active hits in comparison with the standard drug are summarized (Table 1).

**Table 1:** Docking Score of the Top 5 Screened Compounds and Their Protein-Ligand Residual Interactions

sr., no.	ligand ID	docking score (kcal/mol)	amino acid residues
1.	Vildagliptin	–7.58	ARG-125, GLU-205, GLU-206, HIS740
2.	EOS34295	–9.218	ASN-710, ARG-125, SER-630, GLU-205, GLU-206
3.	EOS4915	–8.413	TYR-666, TYR-631, TYR-547, GLY-632
4.	EOS9480	–10.278	TYR-631, TYR-547, GLU-205, GLU-206, ARG-125, PHE-357
5.	EOS2567	–10.548	TYR-666, ASN-710, ARG-125, GLU-205, GLU-206
6.	EOS62725	–12.266	TYR-666, TYR-662, ARG-125, GLU-205, GLU-206

Comparable observations have been reported in previous computational studies targeting DPP-IV. Tsahnang Fofack et al.^8^ identified potential DPP-IV inhibitors through virtual screening and molecular dynamics approaches and reported that favorable inhibitory activity was associated with stable occupancy of the catalytic cavity and interactions with residues involved in substrate recognition.^40,41^ In agreement with those findings, the present study identified compounds exhibiting stronger predicted binding affinities than vildagliptin and occupying the same active pocket. Notably, residues ARG125, GLU205, and GLU206 observed in the present work are recognized as important catalytic and ligand-recognition residues in DPP-IV inhibition studies, supporting the biological relevance of the identified binding modes.

Detailed interaction analysis revealed that all top-ranking compounds formed multiple key noncovalent interactions within the active site. These interactions included hydrogen bonding, hydrophobic contacts, π–π stacking, and salt-bridge formation. Critical residues consistently involved in ligand binding included TYR662, TYR666, TYR631, TYR547, ARG125, ASN710, PHE357, GLY632, GLU205, and GLU206. These residues were common between the standard ligand and the test hits, further validating their binding relevance (Figure 2). Overall, the docking analysis demonstrates that all selected compounds successfully bind within the DPP-IV active site and provides a molecular basis for their potential inhibitory activity.

**Figure 2:**
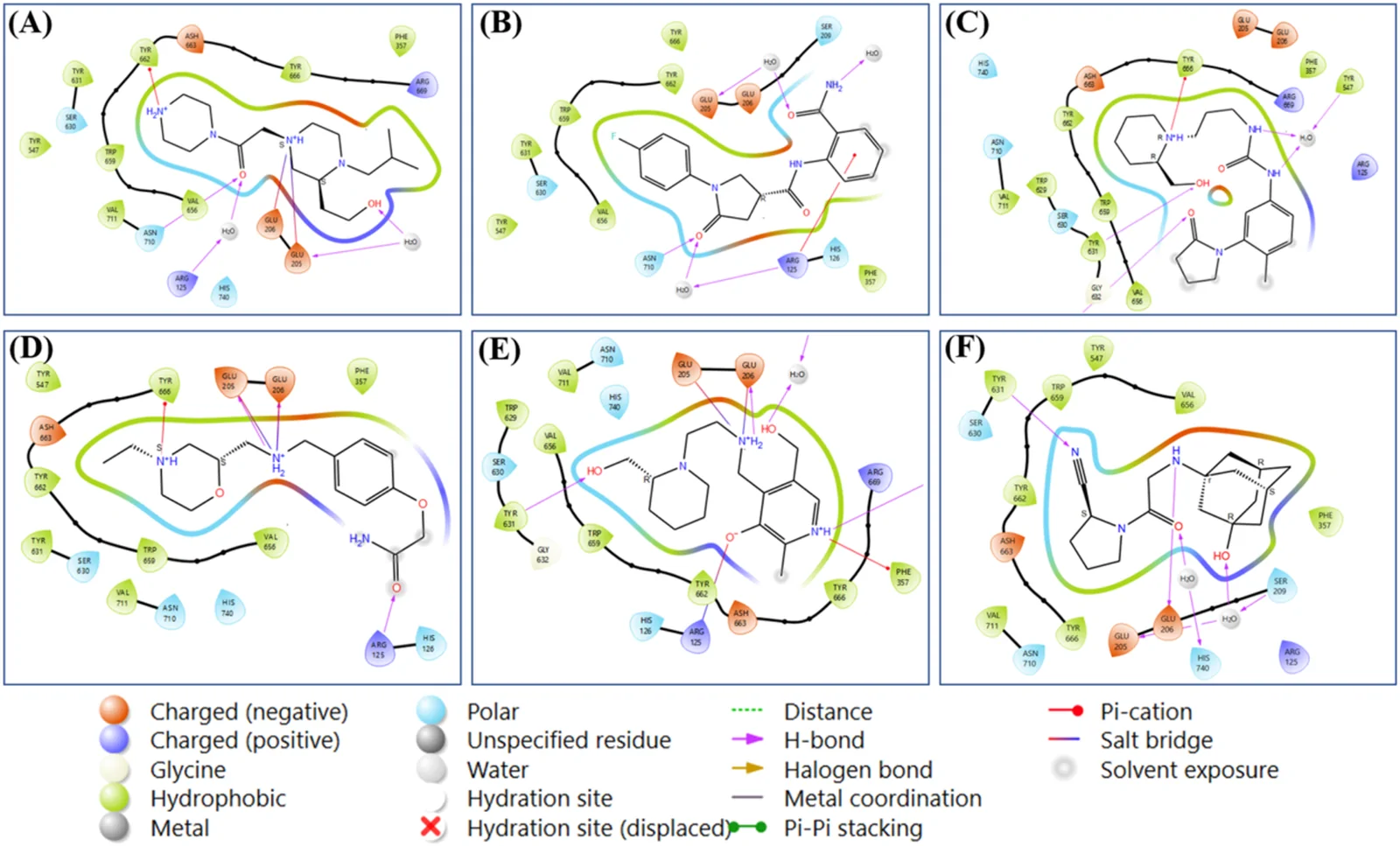
2D representative interaction ligplot profiles of DPP-IV (PDB ID: 6B1E)^29^ with the standard drug vildagliptin and the top five ligand complexes: (A) EOS2567, (B) EOS34295, (C) EOS4915, (D) EOS62725, (E) EOS9480, and (F) Vildagliptin.

Furthermore, the interaction profiles of EOS9480, EOS2567, and EOS62725 suggest a more extensive interaction network than the reference inhibitor, particularly through additional contacts involving TYR662, ASN710, and PHE357. Similar interaction patterns have been associated with enhanced stabilization and improved inhibitory potential in previously reported DPP-IV inhibitor complexes, indicating that the identified hits may possess favorable binding characteristics for further optimization.

#### 3.1.1 Structural Relevance of Identified Chemical Scaffolds with Reported DPP-IV Inhibitors

Beyond the docking and interaction analyses, an additional structural comparison was performed to examine whether the identified lead compounds share chemical features with previously reported DPP-IV inhibitors.^42^ Structural scaffold analysis revealed that several of the top-ranked compounds contain heterocyclic moieties that are frequently observed in marketed DPP-IV inhibitors and experimentally investigated DPP-IV inhibitor series.

Among the identified leads, EOS2567 contains a piperazine moiety, a privileged scaffold widely associated with DPP-IV inhibition.^43–49^ Notably, clinically approved DPP-IV inhibitors including sitagliptin,^44,45^ evogliptin,^46,47^ and teneligliptin^48,49^ incorporate piperazine-containing architectures, where this heterocycle contributes to favorable molecular recognition, conformational adaptability, and interaction with residues surrounding the catalytic cavity. In addition, several previously reported experimental DPP-IV inhibitor series have employed piperazine-containing frameworks to improve inhibitory potency and pharmacokinetic behavior. The presence of this scaffold in EOS2567 therefore supports its structural relevance as a potential DPP-IV inhibitor candidate.

Similarly, EOS34295 contains a pyrrolidine ring, which represents another well-established pharmacophore among DPP-IV inhibitors. The marketed inhibitor vildagliptin contains a pyrrolidine-based framework and exerts potent inhibition through favorable accommodation within the catalytic region of DPP-IV. Pyrrolidine derivatives^50–52^ have also been extensively explored in experimental and medicinal chemistry studies targeting DPP-IV because of their ability to promote stable protein–ligand interactions and enhance binding selectivity. The identification of this scaffold in EOS34295 suggests structural compatibility with known DPP-IV inhibitor design principles.

Furthermore, EOS62725 contains a morpholine moiety, another heterocyclic scaffold that has attracted interest in antidiabetic drug discovery. Morpholine-containing DPP-IV inhibitors^53^ have been investigated in multiple studies due to their balanced physicochemical properties, hydrogen-bonding capability, and favorable oral drug-like characteristics. The occurrence of this scaffold in EOS62725 may contribute to the favorable docking performance and dynamic stability observed during simulation.

Collectively, the recurrence of piperazine, pyrrolidine, and morpholine-containing frameworks among the identified lead compounds strengthens the biological plausibility of the screening outcomes and indicates that the computational workflow successfully enriched chemical features previously associated with DPP-IV inhibitory activity. Although experimental validation is still required, the observed scaffold similarity provides additional support for prioritizing these compounds for future biological evaluation.

### 3.2 Docking Validation

We performed the redocking to validate the docking parameters and ensure the reliability of the docking protocol within the active site of the target protein DPP-IV depicted in Figure 3. Evaluating molecular precision and stability is a critical characteristic of this analysis. This approach helps verify that the docking parameters can accurately reproduce the observed binding pose and affinity, thus reinforcing the credibility of the results. The binding affinities of the top hit compounds showed slight fluctuations in docking score, further confirming the reproducibility and robustness of the docking procedure.

**Figure 3:**
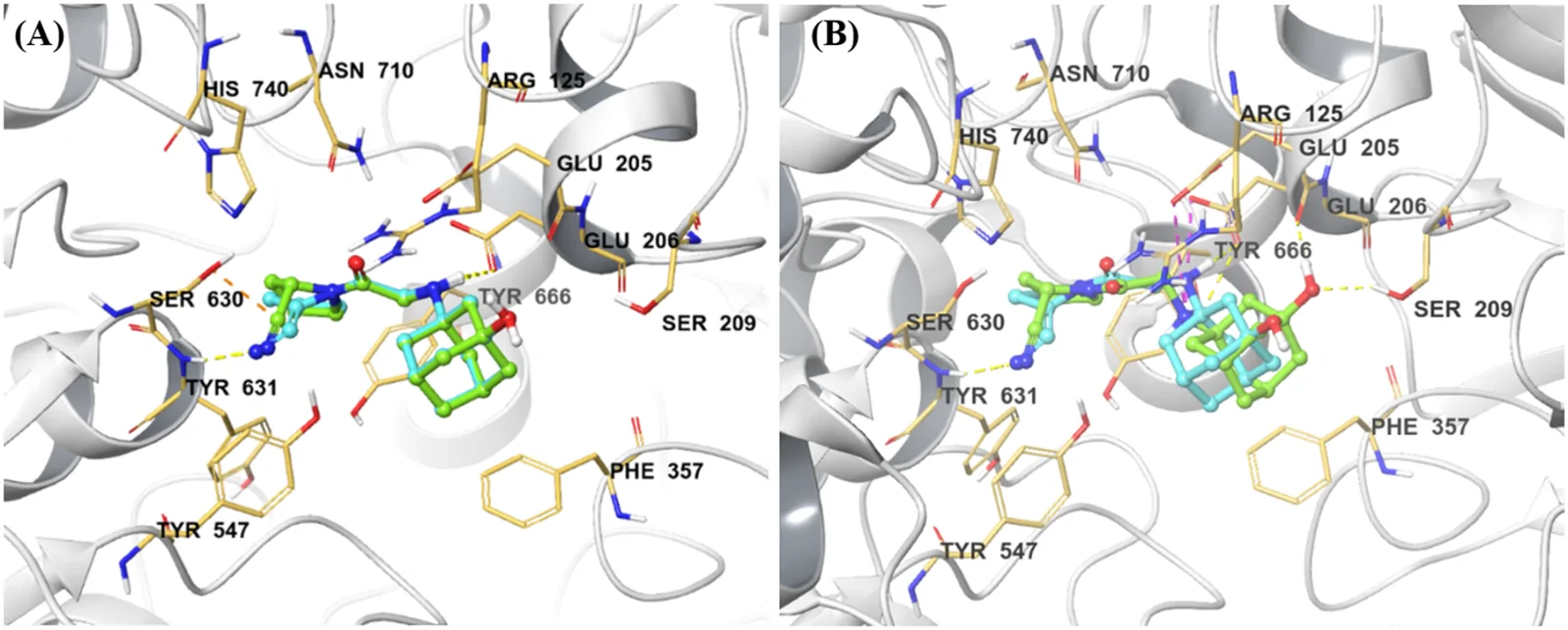
Regeneration and validation of vildagliptin structure for docking in DPP-IV (Chain A): (A) Structural regeneration of vildagliptin to address the unresolved SER680 region in Chain A of the crystal structure. The initial regenerated ligand (cyan) shows the original orientation prior to optimization, while hydrogen-bond refinement and restrained minimization yield a slightly adjusted pose (green), particularly in the cyano and pyrrole groups. This optimized structure was used for grid generation and downstream docking. (B) Redocked vildagliptin (green) superimposed with the optimized ligand pose (cyan), demonstrating close alignment and confirming the reliability of the docking protocol within the DPP-IV active site.

### 3.3 ADMETox Analysis

To further strengthen the pharmacokinetic evaluation, in silico ADME profiling of the top five screened hits was additionally performed using the SwissADME web server, and in-silico toxicity assessment was conducted using ProTox-III. The results are summarized in Tables S1 and S2. Additionally, the SwissADME bioavailability radar plots were generated to visually assess the oral drug-likeness space of the selected compounds (Figure S1).

The physicochemical parameters predicted by SwissADME indicated that all five compounds EOS2567, EOS4915, EOS9480, EOS34295, and EOS62725 possess favorable drug-like characteristics. The molecular weights ranged from 307.39 to 388.50 Da, remaining well within the acceptable limit (<500 Da). All compounds exhibited zero Lipinski rule violations, confirming their suitability for oral drug development. The bioavailability score was consistently predicted as 0.55 for all candidates, indicating good oral bioavailability potential. The fraction of sp^3^ carbons (Csp^3^), an indicator of molecular complexity and three-dimensional character, varied between 0.17 and 0.94, with EOS2567 showing the highest saturation level (0.94), suggesting favorable structural flexibility. Hydrogen bond acceptor (HBA) values ranged from 4–6, while hydrogen bond donor (HBD) values ranged from 2–4, both within optimal drug-like ranges. Topological polar surface area (TPSA) values (59.05–92.50 Å^2^) further supported good membrane permeability and oral absorption characteristics. Lipophilicity (iLOGP) values ranged from 1.87 to 3.11, indicating balanced hydrophilic–lipophilic properties without excessive hydrophobicity. Gastrointestinal (GI) absorption was predicted to be high for all compounds except EOS2567, which showed low predicted absorption. None of the compounds were predicted to be blood–brain barrier (BBB) permeant, which may be advantageous in minimizing central nervous system-related adverse effects for antidiabetic therapy. All five compounds were predicted to be P-glycoprotein (P-gp) substrates, suggesting potential influence on efflux transport mechanisms. The SwissADME bioavailability radar plots further confirmed that the selected compounds largely fall within the optimal physicochemical space for oral bioavailability. EOS2567 and EOS62725 remained predominantly within the recommended region, with minor deviations in flexibility. EOS4915 and EOS9480 showed well-balanced distributions across lipophilicity, size, and polarity parameters, supporting their predicted high gastrointestinal absorption. EOS34295 exhibited a slight deviation in the unsaturation (INSATU) parameter, although the remaining descriptors were within acceptable limits. Overall, the radar analysis substantiates the favorable drug-likeness characteristics of the selected leads.

Importantly, none of the compounds were predicted to inhibit major cytochrome P450 isoforms (CYP1A2, CYP2C19, CYP2C9, and CYP3A4), indicating a lower probability of metabolic drug–drug interactions. Skin permeability (log Kp) values ranged between −7.42 and −8.19 cm/s, consistent with acceptable permeability characteristics. In-silico toxicity profiling (Table S2) revealed that none of the compounds were predicted to be hepatotoxic, cardiotoxic, mutagenic, or cytotoxic. Carcinogenicity was predicted only for EOS34295, while all other compounds were classified as noncarcinogenic. Respiratory toxicity was predicted as active for all compounds, and some compounds showed predicted neurotoxicity or nephrotoxicity (EOS4915). However, predicted LD_50_ values indicated varying toxicity classes, with EOS9480 showing the highest predicted LD_50_ value (5900 mg/kg), suggesting relatively lower acute toxicity compared to the other candidates.

The predicted pharmacokinetic behavior observed in the present study is consistent with previously reported characteristics of clinically successful DPP-IV inhibitors, which generally exhibit favorable oral bioavailability, compliance with Lipinski’s rule of five, and balanced lipophilicity to support systemic exposure. The absence of major CYP inhibition among most selected compounds is particularly encouraging, as reduced interference with CYP-mediated metabolism has been highlighted as an important consideration in minimizing drug–drug interactions during antidiabetic therapy development. Similar ADMET trends have been reported in recent computational screening studies of DPP-IV inhibitors, supporting the translational potential of the identified compounds.^8,40,41^

Overall, the expanded ADME and toxicity evaluation confirms that the majority of the identified lead compounds exhibit favorable drug-likeness, acceptable pharmacokinetic behavior, and manageable toxicity profiles, further supporting their potential as promising DPP-IV inhibitor candidates.

### 3.4 Molecular Dynamic Simulation

Based on the docking results, molecular dynamic simulation studies were performed for compatibility, stability, conformational strength, and dynamic behavior of protein–ligand complexes at the scale of atom.^40,41^ The top Glide-XP docked compounds, having significant nonbonded interactions and binding affinity between the protein and ligand complex, was subjected to MD simulation for a duration of 100 ns. The results of the MD simulation are represented as root means square deviation (RMSD), root-mean-square fluctuation (RMSF), and other properties of protein and ligand complexes.

#### 3.4.1 Root Mean Square Deviation (RMSD) Analysis

In the present study, MD simulations of 100 ns duration were conducted for the DPP-IV enzyme in complex with the top-ranked hit compounds (EOS34295, EOS4915, EOS9480, EOS2567, and EOS62725) to elucidate the structural stability and conformational behavior of the protein–ligand assemblies. The RMSD analysis was employed to track the temporal evolution of atomic positional deviations and to quantitatively evaluate the overall conformational stability of each complex throughout the simulation trajectory. Figure 4 depicts the RMSD trajectories of the DPP-IV enzyme and its ligand-bound complexes across a 100 ns MD simulation. The RMSD values for the Cα atoms were computed with respect to the initial coordinates to quantify global structural deviations and assess the conformational stability and equilibration behavior of each system. All DPP-IV–ligand complexes reached equilibrium within the first 5 ns of the trajectory, indicating rapid system relaxation.

**Figure 4:**
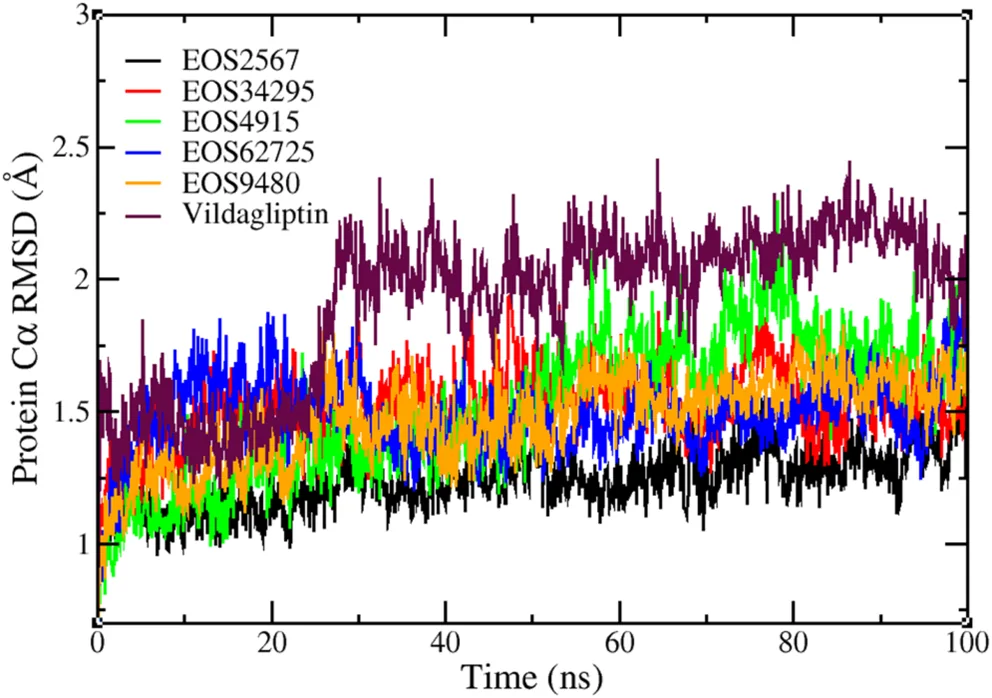
Desmond’s MD simulation analysis showing protein DPP-IV (PDB ID: 6B1E) Cα RMSD plots in complex with ligands (EOS2567, EOS34295, EOS4915, EOS62725, EOS9480, and Vildagliptin) for 100 ns.

The reference DPP-IV–Vildagliptin complex exhibited RMSD fluctuations between 1.4 and 2.25 Å, with noticeable deviations around 25 and 50 ns, suggesting intrinsic flexibility or transient conformational rearrangements. The complex stabilized during the midsimulation phase (50–90 ns), converging to an average RMSD of 1.90 Å, reflecting acceptable structural stability. The DPP-IV–EOS2567 complex showed a smooth and stable RMSD profile throughout the simulation, with only minor perturbations between 60 and 80 ns, where RMSD briefly reached 1.4 Å. The complex maintained an average RMSD of 1.20 Å, indicative of strong binding affinity and stable conformational integrity within the active site. Similarly, the DPP-IV–EOS34295 complex demonstrated a well-equilibrated RMSD trajectory with minimal deviations across the entire 100 ns simulation. A transient elevation around 50–75 ns, peaking at 1.95 Å, was observed; however, the system remained stable overall, sustaining an average RMSD of 1.51 Å, signifying robust ligand–protein interactions and restricted conformational drift.

The DPP-IV–EOS4915 complex displayed minor RMSD perturbations around 20, 50, 70, and 80 ns, with values ranging from 0.5 to 2.4 Å and a maximum deviation of 2.5 Å. Following these transient fluctuations, the system attained stability with an average RMSD of 1.51 Å, suggesting that despite brief conformational adjustments, the protein–ligand complex stabilized effectively during the latter half of the simulation. In contrast, the DPP-IV–EOS62725 complex exhibited consistently low RMSD values throughout the trajectory, apart from a slight fluctuation between 20 and 86 ns, where RMSD temporarily reached 1.75 Å. The average RMSD of 1.48 Å confirms the overall conformational steadiness and favorable interaction profile of EOS62725 within the DPP-IV active pocket. The DPP-IV–EOS9480 complex showed RMSD values ranging from 0.92 to 1.72 Å, with minor deviations at 25, 60, and 85 ns. The system maintained an average RMSD of 1.47 Å and displayed a progressive stabilization trend toward the final 100 ns, indicative of moderate conformational adaptability likely associated with local rearrangements within the binding cavity.

The observed RMSD behavior is consistent with previous molecular dynamics studies of DPP-IV inhibitor complexes,^40,41^ where RMSD values below approximately 2.0 Å were generally interpreted as indicators of stable ligand accommodation and preserved structural integrity of the enzyme. Compared with earlier reports that described moderate structural adaptation following ligand binding, the present hit compounds maintained lower average RMSD values, suggesting improved stabilization of the DPP-IV catalytic environment and stronger conformational persistence during simulation.

Overall, the MD simulations revealed that all complexes, including the reference DPP-IV–Vildagliptin and the newly identified hit compounds, preserved stable conformations throughout the 100 ns simulation. Notably, the consistently low RMSD values of the hit complexes (1.20–1.51 Å) compared with Vildagliptin (1.90 Å) demonstrate enhanced conformational stability of the enzyme upon ligand binding, underscoring the strong interaction potential and promising inhibitory characteristics of EOS34295, EOS4915, EOS9480, EOS2567, and EOS62725 against DPP-IV.

#### 3.4.2 Root Mean Square Fluctuation (RMSF) Analysis

Root mean square fluctuation provides a residue-level assessment of structural flexibility and dynamic behavior throughout MD simulation. This metric is particularly valuable for identifying regions of enhanced mobility within a protein that may influence ligand recognition, binding stability, and overall conformational integrity. In the present study, RMSF calculations were performed on the Cα atoms of the DPP-IV protein complexed with selected ligands (EOS2567, EOS34295, EOS4915, EOS62725, and EOS9480) and standard drug vildagliptin over a 100 ns simulation length (Figure 5).

**Figure 5:**
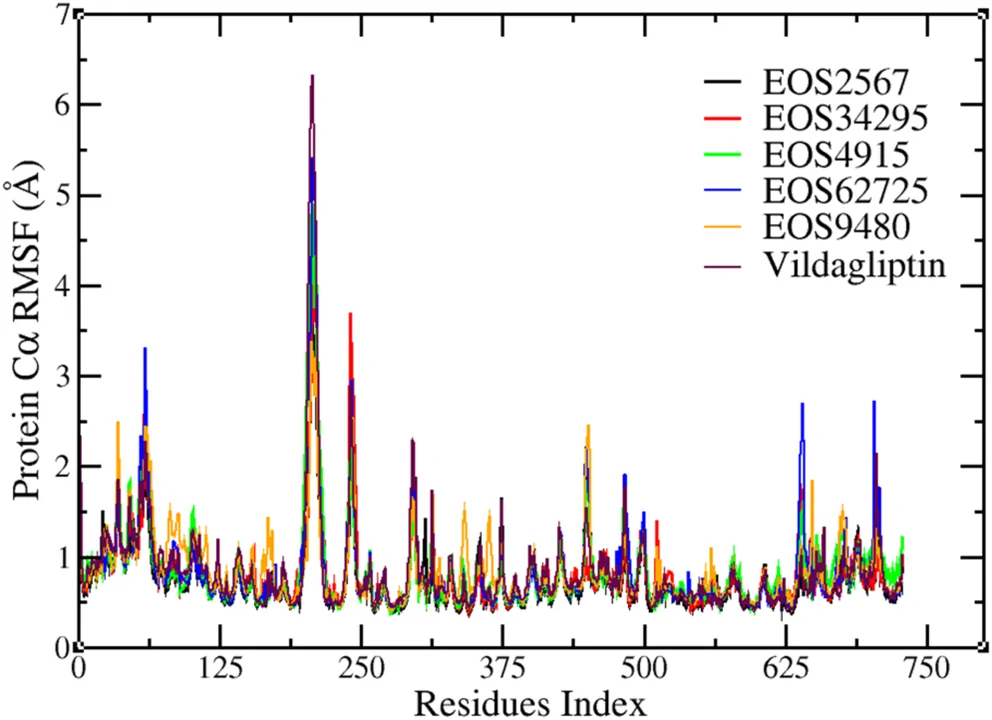
Desmond’s MD simulation analysis showing protein DPP-IV (PDB ID: 6B1E) (Cα) RMSF plots in complex with ligands (EOS2567, EOS34295, EOS4915, EOS62725, EOS9480, and Vildagliptin) for 100 ns.

For the native cocrystallized DPP-IV–Vildagliptin complex, the average RMSF was 0.86 Å. Pronounced flexibility was observed in the TYR241–ASP243, GLU244–LEU246, GLN247–PRO249, and SER277–SER278 segments, with peak fluctuations of 5.73 Å, 6.20 Å, 4.72 Å, and 3.02 Å, respectively. These elevated deviations indicate that these regions possess inherent mobility that may contribute to structural adaptation during ligand binding. In the DPP-IV–EOS2567 complex, the average RMSF (0.74 Å) was slightly lower than that of the reference drug, suggesting improved stabilization. The formation of persistent hydrogen bonds with GLU205 and GLU206 contributed significantly to the reduced residue-level flexibility (Figure 5). Although GLU244 (3.23 Å), SER245 (3.62 Å), and GLN247 (3.22 Å) exhibited moderate fluctuations, the overall profile demonstrated lower mobility relative to the DPP-IV–Vildagliptin complex, indicating that EOS2567 enhanced structural rigidity within the active site. The DPP-IV–EOS34295 complex showed an average RMSF of 0.80 Å, with localized fluctuations in GLU244 (2.94 Å), SER245 (4.24 Å), LEU246 (3.60 Å), GLN247 (3.22 Å), and TYR248 (3.49 Å). These slight deviations reflect typical loop dynamics while maintaining global structural stability upon ligand engagement.

For the DPP-IV–EOS4915 complex, the average RMSF was 0.83 Å. Modest fluctuations were detected in residues PHE240 (3.18 Å), TYR241 (3.14 Å), SER242 (3.70 Å), ASP243 (3.91 Å), GLU244 (4.77 Å), SER245 (4.81 Å), LEU246 (3.73 Å), GLN247 (4.40 Å), and TYR248 (3.62 Å). These variations are characteristic of flexible loop motifs undergoing conformational adaptation during ligand interaction. The DPP-IV–EOS62725 complex maintained an average RMSF of 0.83 Å. Notable fluctuations were observed in the LEU97–PHE98 (3.30 Å), ASP243–SER245 (5.41 Å), LEU246–TYR248 (4.30 Å), and SER278–VAL279 (2.85 Å) regions, indicating moderate mobility while preserving overall structural stability. The DPP-IV–EOS9480 complex exhibited an average RMSF of 0.86 Å. Localized peaks at ASP243 (2.72 Å), GLU244 (3.38 Å), SER245 (3.28 Å), LEU246 (2.78 Å), and GLN247 (3.20 Å) indicate flexibility consistent with the dynamic loop region frequently observed in other complexes.

Residue-specific flexibility patterns observed in this work are also consistent with previously reported DPP-IV simulation studies,^40,41^ where loop regions surrounding residues 240–250 frequently exhibit greater mobility than the catalytic core. Importantly, the absence of significant fluctuations near the active-site residues indicates that ligand binding did not disrupt catalytic architecture, reinforcing the structural compatibility of the identified compounds with the DPP-IV binding pocket.

Overall, the RMSF profiles demonstrated similar patterns of localized flexibility among all ligand-bound complexes when compared with the reference DPP-IV–Vildagliptin system. Residues contributing to dynamic behavior including ASP243, GLU244, SER245, LEU246, GLN247, and TYR248 were consistently observed across all trajectories. Importantly, the relatively low RMSF magnitudes and restricted fluctuations indicate that new ligand binding did not induce destabilizing flexibility; instead, the hit compounds contributed to maintaining or enhancing the conformational stability of DPP-IV throughout the 100 ns MD simulation.

#### 3.4.3 SASA and Radius of Gyration

To further investigate the structural stability and compactness of the DPP-IV protein during the molecular dynamics simulations, the solvent-accessible surface area (SASA) and radius of gyration (Rg) of the protein within the protein–ligand complexes were analyzed (Figures 6 and[Fig fig7]). The radius of gyration provides information on the overall compactness and structural integrity of the protein during the simulation trajectory, whereas SASA reflects the degree of solvent exposure of the protein surface.

**Figure 6:**
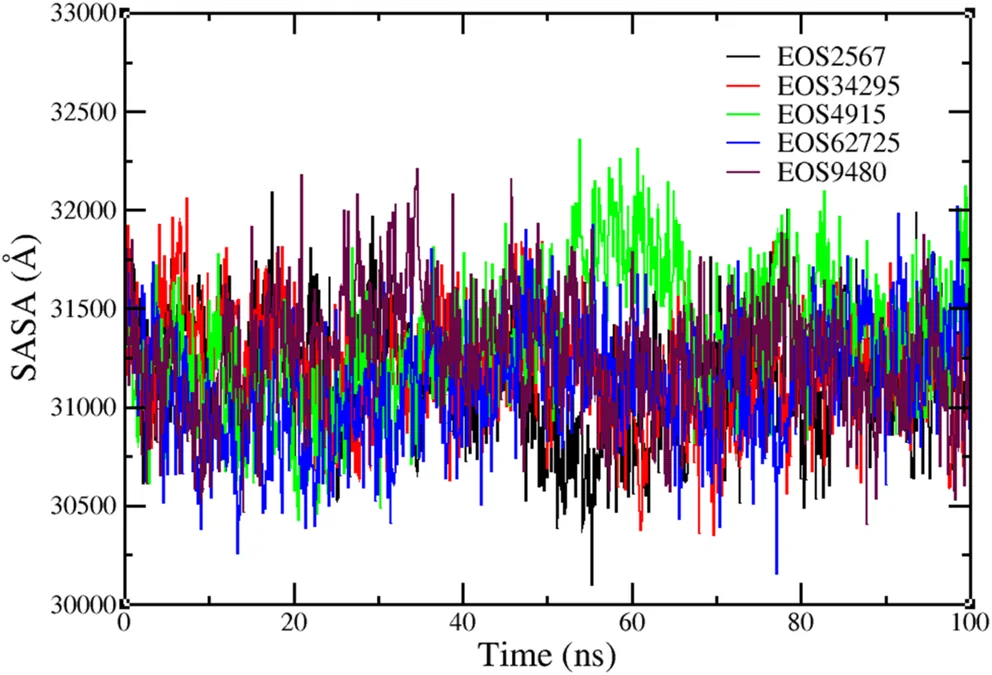
Time evolution of the SASA of the DPP-IV protein in complex with EOS2567, EOS34295, EOS4915, EOS62725, and EOS9480 during the 100 ns molecular dynamics simulation.

**Figure 7:**
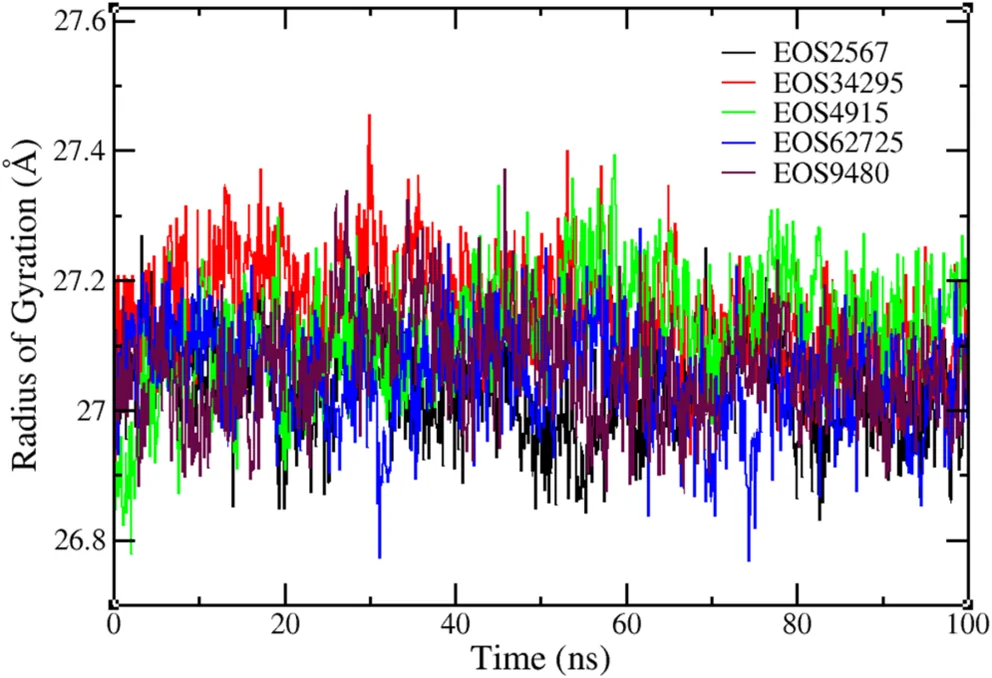
Time evolution of the Rg of the DPP-IV protein in complex with EOS2567, EOS34295, EOS4915, EOS62725, and EOS9480 during the 100 ns molecular dynamics simulation.

Comparable observations have been reported in previous MD-based investigations of DPP-IV inhibitors,^40,41^ where lower SASA and stable radius of gyration values were associated with improved protein compactness and sustained ligand binding. The relatively lower fluctuations observed for EOS2567, EOS34295, EOS62725, and EOS9480 suggest favorable structural stabilization, whereas the slightly elevated values for EOS4915 may reflect greater conformational adaptability during protein–ligand accommodation.

As shown in Figure 6, the SASA profiles of the DPP-IV complexes with EOS2567, EOS34295, EOS4915, EOS62725, and EOS9480 remained relatively stable throughout the 100 ns simulation, exhibiting only minor fluctuations. The relatively consistent SASA values suggest that ligand binding did not induce major structural perturbations in the protein and that the complexes maintained stable solvent exposure during the simulation. However, the complex with EOS4915 exhibited slightly higher SASA values compared to the other ligands, indicating relatively greater solvent exposure of the protein surface in this system.

Similarly, the radius of gyration values of the protein in all complexes remained largely stable over the simulation period (Figure 7), indicating that the overall compactness of the DPP-IV structure was generally preserved. Nevertheless, the DPP-IV–EOS4915 complex showed comparatively higher Rg values, suggesting a slightly less compact protein conformation relative to the other ligand-bound systems. The increased SASA and Rg observed for EOS4915 may indicate relatively weaker stabilization of the protein structure and reduced compactness compared with the other ligands, which could reflect comparatively lower binding stability during the simulation.

In contrast, the remaining complexes exhibited relatively lower and stable SASA and Rg values, suggesting that these ligands maintained better structural compactness of the protein and may contribute to stronger stabilization of the DPP-IV structure during the simulation. The small variations observed in the Rg profiles are typical of stable proteins undergoing natural conformational motions during molecular dynamics simulations.

In addition, ligand-specific structural parameters were analyzed to further evaluate ligand behavior and stability within the DPP-IV binding pocket during the molecular dynamics simulations. These analyses included ligand Rg, molecular surface area (MolSA), SASA, and polar surface area (PSA), which provide insights into ligand compactness, surface exposure, and interaction potential throughout the simulation trajectory. As shown in Figure S3, these parameters remained generally stable for all ligand systems, indicating that the ligands maintained consistent conformations and surface characteristics while interacting with the DPP-IV active site. Minor fluctuations observed in these profiles reflect natural dynamic adjustments of the ligands within the binding pocket during the simulation. Furthermore, ligand torsional angle distributions were examined to assess the rotational flexibility of key bonds. The torsional profiles presented in Figure S4 indicate that most rotatable bonds occupy preferred dihedral angle regions with limited transitions between conformational states, suggesting restricted rotational flexibility and stable ligand orientations during the simulation.

#### 3.4.4 Post MD Protein–Ligand Interactions Analysis

Monitoring protein–ligand detailed interactions over the 100 ns MD simulation revealed the contribution of individual residues to complex stabilization. The interaction diagrams of DPP-IV complexes (Figures 8 and 9) showed that binding was primarily mediated by hydrogen bonds and hydrophobic forces.

**Figure 8:**
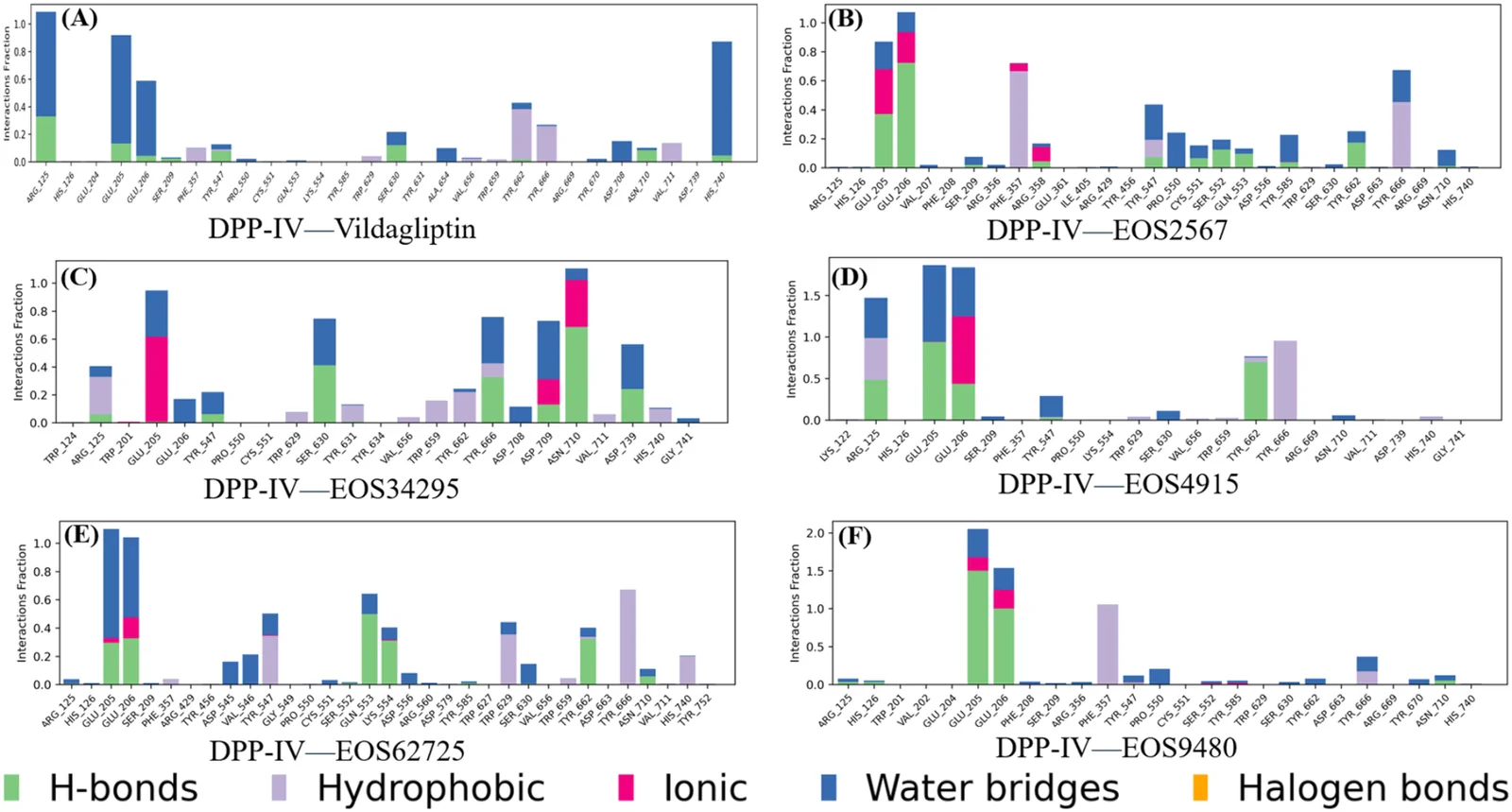
2D interaction fraction diagrams of Vildagliptin, EOS2567, EOS34295, EOS4915, EOS62725, and EOS9480 in complex with DPP-IV, illustrating the interaction fractions of key amino-acid residues throughout the 100 ns MD simulation. The diagrams represent the relative stability and frequency of hydrogen bonds, hydrophobic contacts, *π–π* interactions, and other noncovalent interactions maintained by each ligand during the simulation.

**Figure 9:**
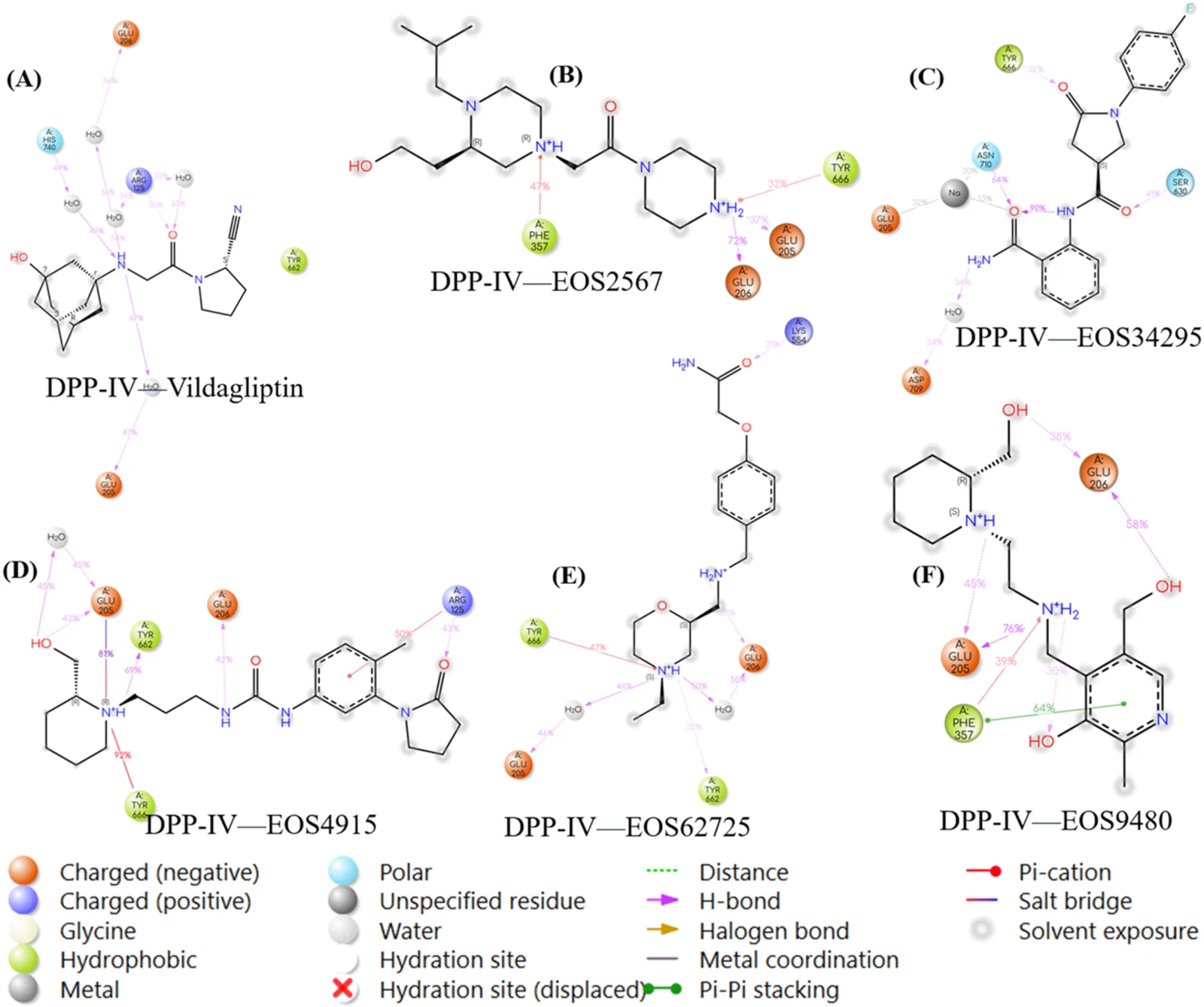
2D interaction diagram of Vildagliptin, EOS2567, EOS34295, EOS4915, EOS62725, and EOS9480 in complex with DPP-IV throughout the MD simulation (100 ns).

In the reference DPP-IV–vildagliptin complex, stable hydrogen bonds were observed (Figures 8 and 9) with ARG125, GLU205, GLU206, and TYR666. These interactions were preserved and further strengthened in the hybrid ligand complexes (QEOS2567, EOS34295, EOS4915, EOS62725, and EOS9480), which also involved additional residues such as PHE357, LYS554, SER630, TYR662, ASP709, and ASN710 suggesting enhanced binding compared to the reference inhibitor. The most significant hydrophobic interactions were contributed by GLU205, GLU206, and TYR666, which consistently stabilized the ligand throughout the 100 ns simulation. Notably, QEOS2567, EOS34295, EOS4915, EOS62725, and EOS9480 maintained these conserved interactions over the full trajectory.

Interestingly, additional hydrogen bond with PHE357 were observed in the hit compounds during MD simulations but were not detected in the initial docking study. These results indicate that the hybrid ligands form stronger and more persistent interactions with the DPP-IV active site compared to vildagliptin, supporting their potential as effective DPP-IV inhibitors.

Several computational investigations of DPP-IV inhibitors have highlighted the importance of maintaining interactions with catalytic residues GLU205 and GLU206 together with surrounding recognition residues such as TYR662, TYR666, ARG125, and ASN710 for effective inhibition. Consistent with these reports, the identified compounds preserved these conserved contacts throughout the simulation trajectory and additionally developed persistent interactions with PHE357, which were absent during the initial docking stage. The emergence of these interactions during MD simulation suggests dynamic optimization of ligand accommodation and stronger stabilization within the active site. Compared with previously reported DPP-IV screening studies, the present workflow integrating docking, MD simulation, MM/GBSA, and ADMET profiling provides a more comprehensive prioritization strategy and strengthens confidence in the identified lead candidates.

### 3.5 Binding Free Energy Calculation

The initial binding free energy is a postdocking approach and is a liable and attractive computational technique for the analysis of the equilibrium of ligand-protein complex in a solvent system (VSGB 2.0 solvation system). The Prime-MMGBSA analysis of postdocking and post-MD simulation of protein–ligand complexes was performed, and the Pose Viewer (PV) output obtained from the Glide docking process was used in the Prime MMGBSA module of the maestro, from which the initial binding free energy was calculated. The post-MD simulation binding energy was calculated from the last 50 ns (50–100 ns) of the lead compounds trajectories to evaluate the thermodynamics parameters of the protein–ligand complex such as binding free energy. Our results of Prime-MMGBSA show that the average binding energy for the protein–ligand complex DPP-IV-EOS34295, DPP-IV-EOS4915, DPP-IV-EOS9480, DPP-IVEOS2567, DPP-IV-EOS62725 is −56.5803 (kcal/mol), −48.4880 (kcal/mol), −34.3239 (kcal/mol), −31.2773 (kcal/mol), and −37.9047 (kcal/mol) respectively. We also have calculated the Coulomb energy, van der Waals forces, binding covalent energy, electrostatic energy, polar solvation energies, and lipophilic energy of the complex depicted in Table 2.

**Table 2:** Binding Free Energies (Δ*G*_bind_) of the EOS Series of Compounds Bound with 6B1E Active Site

sr., no.	protein–ligand systems	avg binding free energy from post MD simulation (Δ*G*_bind_) kcal/mol
1.	DPP-IV–Vildagliptin	–23.88 ± 4.52
2.	DPP-IV–EOS34295	–56.58 ± 7.45
3.	DPP-IV–EOS4915	–48.48 ± 5.66
4.	DPP-IV–EOS9480	–34.32 ± 5.34
5.	DPP-IV–EOS2567	–31.27 ± 5.24
6.	DPP-IV–EOS62725	–37.90 ± 7.41

It is important to note that the present study is based on computational approaches, and therefore the identified compounds require further experimental validation to confirm their biological activity. However, such in silico strategies are widely employed in early stage drug discovery to efficiently prioritize candidate molecules from large chemical libraries. The integrated workflow used in this study, combining molecular docking, binding free energy calculations, molecular dynamics simulations, and ADMET analysis, provides a robust framework for identifying stable and drug-like DPP-IV inhibitors. The identified lead compounds may serve as promising candidates for future experimental investigation.

The MM/GBSA results further support the observations obtained from docking and MD simulations. Previous studies investigating DPP-IV inhibitor binding have shown that more negative binding free-energy values are generally associated with enhanced complex stability and stronger predicted inhibitory potential. In comparison with vildagliptin, the identified compounds particularly EOS34295 and EOS4915 displayed substantially more favorable Δ*G*_bind_ values, suggesting improved thermodynamic stabilization within the active site. These findings collectively indicate that the identified compounds may represent promising scaffolds for further experimental validation and optimization.

## 4 Conclusion

In summary, structure-based virtual screening of 5,500 ECBD small molecules was carried out to identify novel inhibitors of the DPP-IV enzyme, a key therapeutic target in glycemic regulation. Molecular docking identified several high-affinity ligands EOS34295, EOS4915, EOS9480, EOS2567, and EOS62725 with docking scores superior to the reference inhibitor vildagliptin and showing strong interactions with essential catalytic and substrate-recognition residues (ARG-125, GLU-205, GLU-206, TYR-631, TYR-662, TYR-666, ASN-710, SER-630, and PHE-357). Pharmacokinetic evaluation further confirmed acceptable ADMETox profiles, indicating their potential suitability as orally bioavailable antidiabetic agents.

In the following step, 100 ns MD simulations provided deeper insight into the dynamic stability and binding behavior of the top complexes. All ligand-bound DPP-IV systems, including the reference vildagliptin complex, maintained stable conformations throughout the simulation period. Notably, the hit compounds exhibited markedly lower RMSD values (1.20–1.51 Å) compared to vildagliptin (1.90 Å), indicating enhanced conformational stabilization of the enzyme upon their binding. This suggests that the newly identified ligands offer stronger and more consistent stabilization of the DPP-IV active site environment. RMSF profiles revealed comparable and localized flexible regions across all systems (including ASP243, GLU244, SER245, LEU246, GLN247, and TYR248), with no destabilizing fluctuations induced by the new ligands. Instead, the hit compounds contributed to maintaining or improving the structural stability of DPP-IV, demonstrating favorable physicochemical complementarity. Importantly, MD trajectory analysis revealed additional persistent hydrogen-bond interactions with PHE357 in the hit compounds interactions that were absent in the initial docking results indicating enhanced stabilization and deeper binding accommodation during the natural dynamic evolution of the complexes. These newly observed interactions highlight the capability of the screened molecules to adapt within the binding pocket and form stronger, more persistent contacts than the reference inhibitor.

Our free energy analysis using thermal MM/GBSA binding free-energy calculations further validated the superior binding strength of the top candidates. EOS34295 (−56.58 ± 7.45 kcal/mol), EOS62725 (−37.90 ± 7.41 kcal/mol), and EOS2567 (−31.27 ± 5.24 kcal/mol) demonstrated significantly stronger binding affinities relative to vildagliptin (−23.88 kcal/mol), confirming their high thermodynamic stability in the active site. Overall, EOS34295, EOS2567, and EOS62725 emerged as the most promising lead molecules across all computational evaluations docking, ADMETox, MD dynamics, and binding free-energy analysis. Their superior stability, strong interaction networks, and favorable energetics collectively support their candidacy as potent, novel DPP-IV inhibitors. Importantly, structural analysis of the identified lead compounds revealed the presence of heterocyclic scaffolds frequently associated with DPP-IV inhibition, including piperazine, pyrrolidine, and morpholine moieties. These structural features are found in several marketed or experimentally investigated DPP-IV inhibitors, such as sitagliptin, evogliptin, teneligliptin, and vildagliptin, providing additional support for the biological relevance of the identified hits. The recurrence of these privileged scaffolds among the top-ranked compounds further strengthens their potential as promising starting points for the development of next-generation DPP-IV inhibitors. However, additional biological studies (in vivo and in vitro) are required to validate these computational findings. Overall, the present results provide a strong foundation for advancing these potential DPP-IV inhibitors toward the development of novel antidiabetic therapeutics.

## Supplementary Material



## Notes

ADME analysis of the top five compounds (Table S1); SwissADME bioavailability radar plots of the top five compounds (Figure S1); in-silico toxicity analysis of the top five compounds (Table S2); 2D chemical structures of the top five lead compounds (Figure S2); molecular dynamics trajectory analysis (RMSD, Rg, intramolecular hydrogen bonds, MolSA, SASA, and PSA) of DPP-IV–ligand complexes (Figure S3); and ligand torsion angle distribution analysis from molecular dynamics simulations (Figure S4) (PDF)
