# Supplementary material for: In Silico Identification of Novel Leads as Potential DPP-IV Inhibitors as Antidiabetic Agents Using Virtual Screening
Source: ACS Omega. 2026 Aug 24;11(35):52439–51. doi: 10.1021/acsomega.5c13626 (PMC13563927; doi:10.1021/acsomega.5c13626)
Supplement: Supplementary file 1 [file ao5c13626_si_001.pdf]

Supplementary Material:

In Silico Identification of Novel Leads as Potential DPP-IV inhibitors as Antidiabetic Agents Using Virtual Screening

Milendra Kumar Turkar[1], Rahul Ahirwar[1], Rishika Sahu[1], Rajendra Bhadane*[2], Surendra Kumar Jain[3], and Deepti Jain*[1]

[1]School of Pharmaceutical Sciences, Rajiv Gandhi Proudyogiki Vishwavidyalaya, Airport Rd, Gandhi Nagar, Bhopal, Madhya Pradesh 462033, India

[2]Institute of Biomedicine, Research Unit for Infection and Immunity, University of Turku, Kiinamyllynkatu 10, Turku FI-20520, Finland

[3]Lakshmi Narain College of Pharmacy, Kalchuri Nagar, Raisen Road, Bhopal - 462021

Table S1: ADME analysis of top five compounds

Ligand ID	Formula	MW	Fraction Csp3	HBA	HBD	MR	TPSA	iLOGP	GI absorption	BBB permeant	Pgp substrate	CYP1A2 inhibitor	CYP2C19 inhibitor	CYP2C9 inhibitor	CYP3A4 inhibitor	log Kp (cm/s)	Lipinski #violations	Bioavailability Score
EOS2567	$C_{16}H_{32}N_4O_2$	312.45	0.94	5	2	103.31	59.05	3.1	Low	No	Yes	No	No	No	No	-8.19	0	0.55
EOS4915	$C_{21}H_{32}N_4O_3$	388.5	0.62	4	3	118.07	84.91	3.11	High	No	Yes	No	No	No	No	-7.72	0	0.55
EOS9480	$C_{16}H_{27}N_3O_3$	309.4	0.69	6	4	89.44	88.85	2.29	High	No	Yes	No	No	No	No	-8.05	0	0.55
EOS34295	$C_{18}H_{16}FN_3O_3$	341.34	0.17	4	2	93.17	92.5	1.87	High	No	Yes	No	No	No	No	-7.42	0	0.55
EOS62725	$C_{16}H_{25}N_3O_3$	307.39	0.56	5	2	87.85	76.82	2.44	High	No	Yes	No	No	No	No	-7.96	0	0.55

Note Table S1*: MW, molecular weight (Da); Fraction Csp³, fraction of sp³-hybridized carbons; HBA, hydrogen bond acceptors; HBD, hydrogen bond donors; MR, molar refractivity; TPSA, topological polar surface area (Å²); iLOGP, predicted lipophilicity (octanol/water partition coefficient); GI absorption, predicted gastrointestinal absorption; BBB permeant, predicted blood–brain barrier permeability; P-gp substrate, P-glycoprotein substrate prediction; CYP1A2, CYP2C19, CYP2C9, CYP3A4 inhibitor, predicted inhibition of respective cytochrome P450 isoforms; log Kp, predicted skin permeability coefficient (cm/s); Lipinski #violations, number of violations of Lipinski's rule of five; Bioavailability Score, predicted oral bioavailability probability score (SwissADME).*

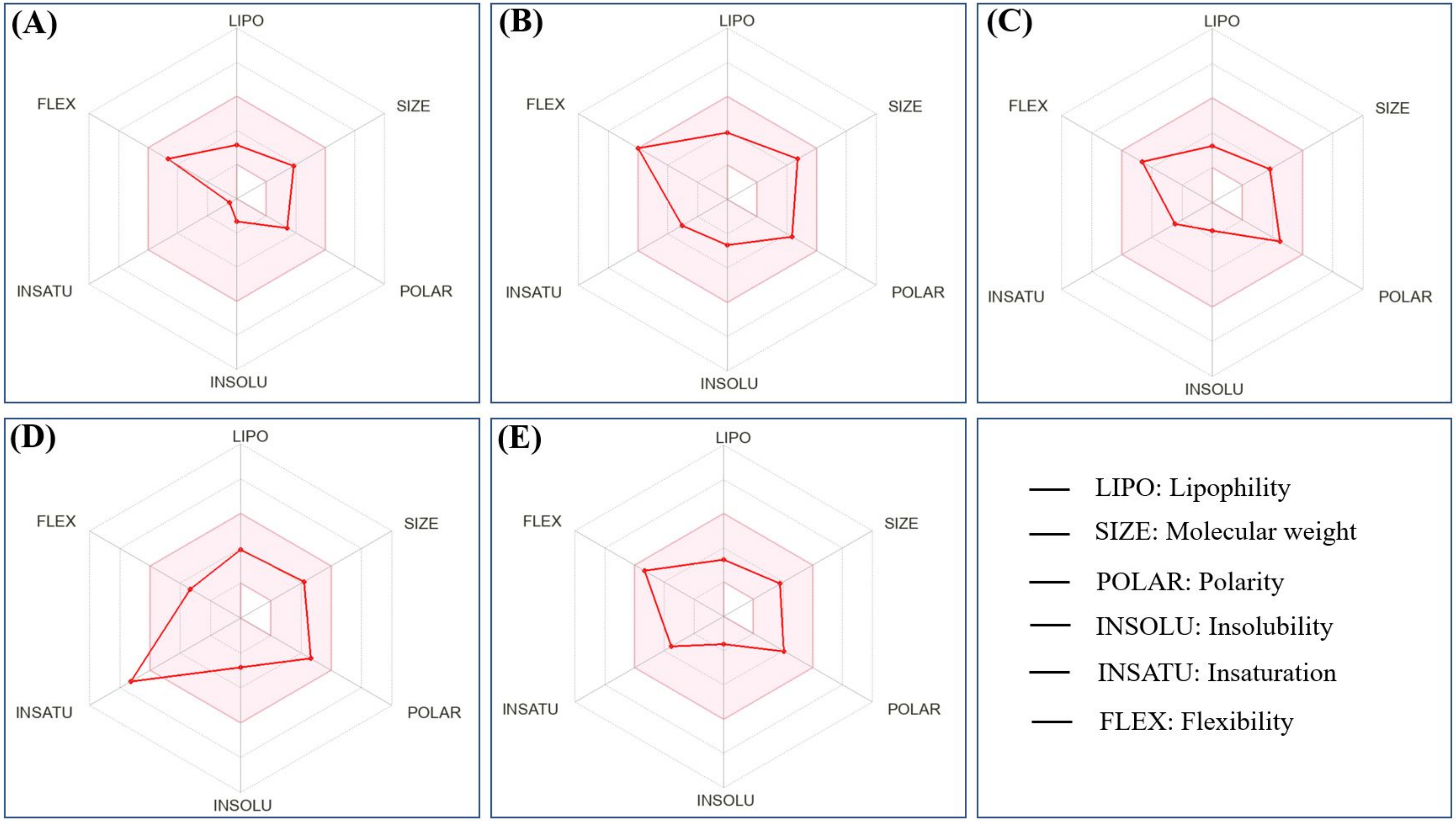

Figure S1: SwissADME bioavailability radar plots illustrating the oral drug-likeness profiles of the top five screened compounds: (A) EOS2567, (B) EOS4915, (C) EOS9480, (D) EOS34295, and (E) EOS62725. The radar representation evaluates six key physicochemical parameters including lipophilicity (LIPO), molecular size (SIZE), polarity (POLAR), solubility (INSOLU), saturation (INSATU), and flexibility (FLEX). The pink shaded region represents the optimal physicochemical space associated with favorable oral bioavailability, while the red polygon indicates the predicted profile of each compound.

Table S2: In-silico toxicity analysis

**Ligand ID**	**Hepatotoxicity**	**Neurotoxicity**	**Nephrotoxicity**	**Respiratory toxicity**	**Cardiotoxicity**	**Immunotoxicity**	**Mutagenicity**	**Cytotoxicity**	**Carcinogenicity**	***CYP1A2***	***CYP2C19***	***CYP3A4***	***p*LD$_{50}$ mg/kg**
EOS2567	Inactive	Inactive	Inactive	Active	Inactive	Inactive	Inactive	Inactive	Inactive	Inactive	Inactive	Inactive	2500
EOS4915	Inactive	Active	Active	Active	Inactive	Inactive	Inactive	Inactive	Inactive	Inactive	Inactive	Inactive	175
EOS9480	Inactive	Active	Inactive	Active	Inactive	Active	Inactive	Inactive	Inactive	Inactive	Inactive	Inactive	5900
EOS34295	Inactive	Active	Inactive	Active	Inactive	Inactive	Inactive	Inactive	Active	Inactive	Inactive	Inactive	1500
EOS62725	Inactive	Active	Inactive	Active	Inactive	Inactive	Inactive	Inactive	Inactive	Inactive	Inactive	Inactive	750

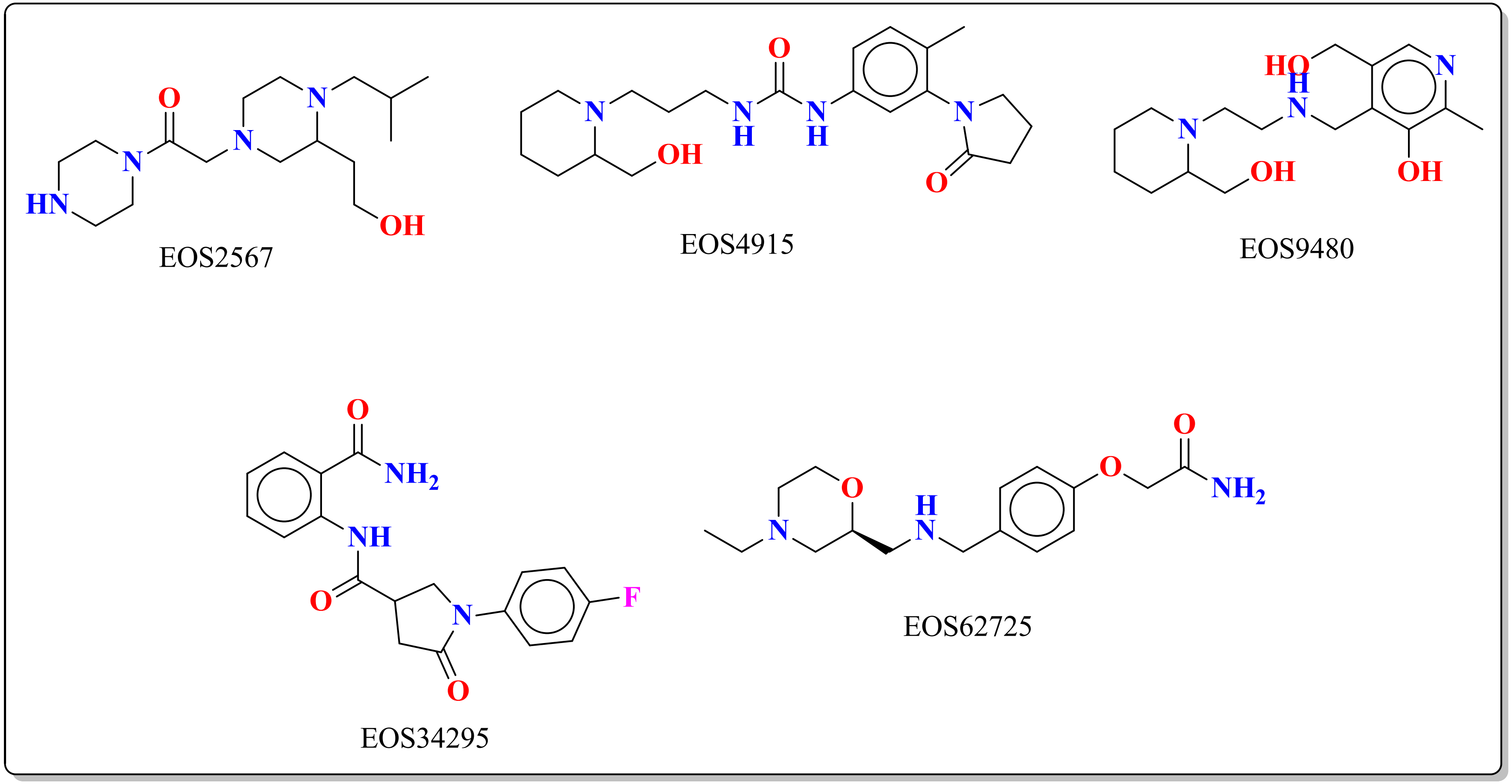

Figure S2: Two-dimensional (2D) chemical structures of the top five selected lead compounds: EOS2567, EOS4915, EOS9480, EOS34295, and EOS62725. The structures illustrate the key functional groups and structural frameworks responsible for hydrogen bonding, hydrophobic interactions, and overall molecular architecture considered during docking, ADME evaluation, and molecular dynamics simulations.

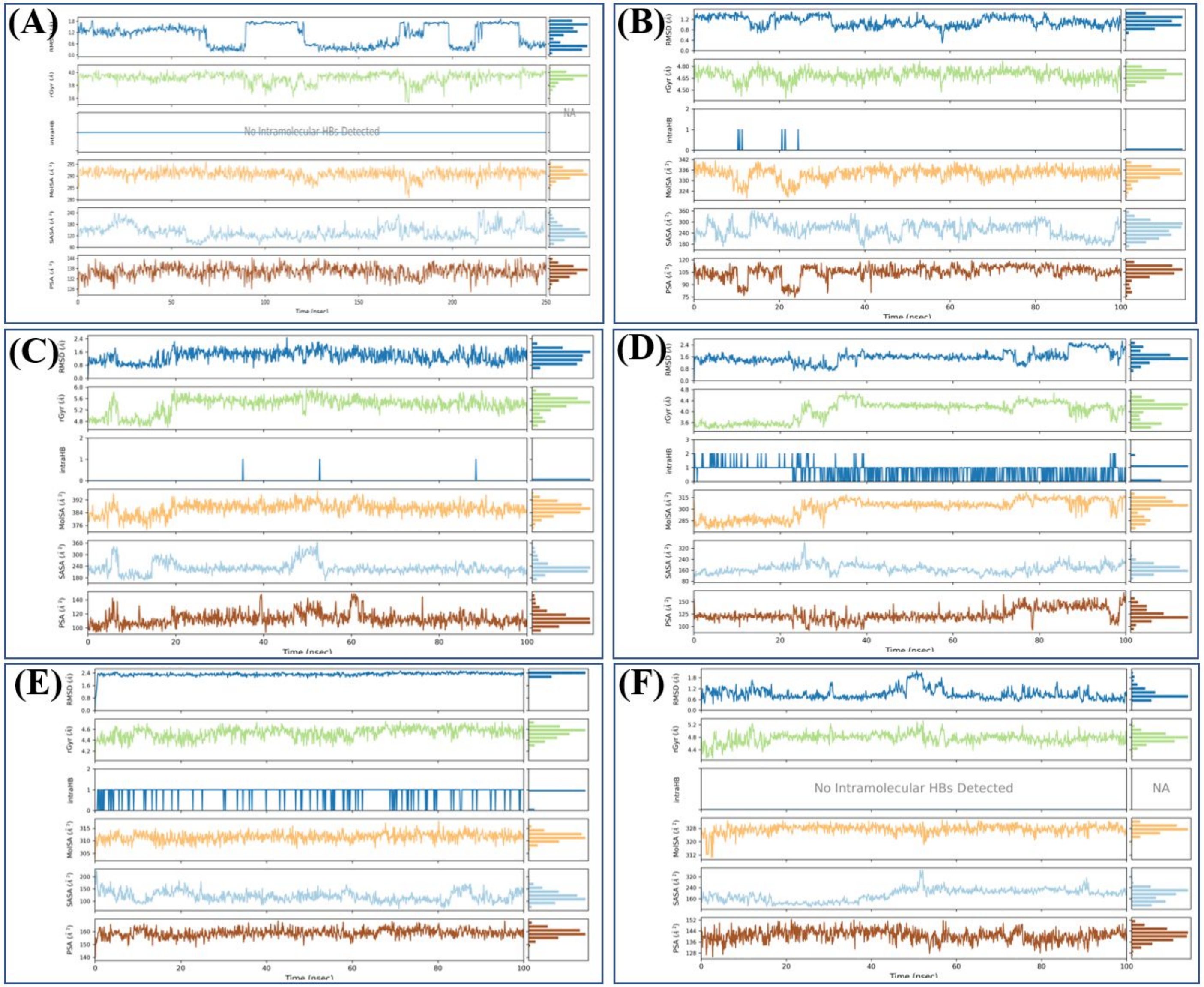

Figure S3: Molecular dynamics simulation analysis of the DPP-IV–ligand complexes over the simulation trajectory. Panels represent the time evolution of key structural parameters including root mean square deviation (RMSD), radius of gyration (Rg), intramolecular hydrogen bonds, molecular surface area (MolSA), solvent-accessible surface area (SASA), and polar surface area (PSA). The analyses are shown for the following complexes: (A) DPP-IV–Vildagliptin (reference inhibitor), (B) DPP-IV–EOS2567, (C) DPP-IV–EOS4915, (D) DPP-IV–EOS9480, (E) DPP-IV–EOS34295, and (F) DPP-IV–EOS62725. These plots illustrate the structural stability, compactness, hydrogen bonding behavior, and conformational dynamics of the protein–ligand complexes throughout the molecular dynamics simulation period.

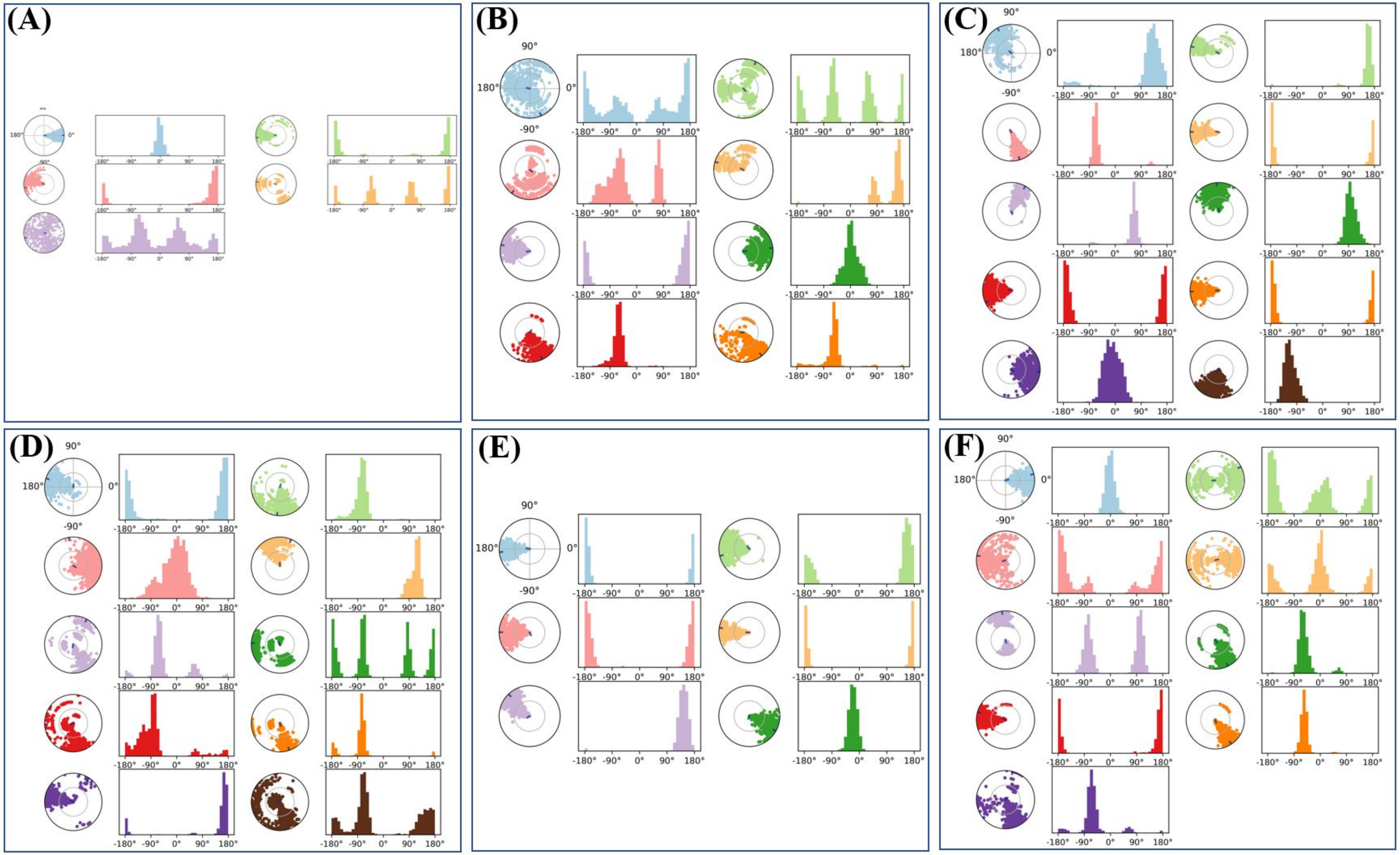

Figure S4: Ligand torsion analysis obtained from molecular dynamics simulations of the DPP-IV–ligand complexes. The plots illustrate the distribution and temporal evolution of ligand torsional angles throughout the simulation trajectory, providing insight into the conformational flexibility and rotational behavior of key bonds within the ligands. Circular plots represent the torsion angle distribution over time, while the accompanying histograms show the frequency distribution of the dihedral angles. The panels correspond to (A) DPP-IV–Vildagliptin (reference inhibitor), (B) DPP-IV–EOS2567, (C) DPP-IV–EOS4915, (D) DPP-IV–EOS9480, (E) DPP-IV–EOS34295, and (F) DPP-IV–EOS62725. The observed torsional profiles provide information on ligand conformational stability and adaptability within the binding pocket during the molecular dynamics simulation.
